# The impact of COVID-19 pandemic on radiology residents in Northern Italy

**DOI:** 10.1007/s00330-021-07740-0

**Published:** 2021-03-23

**Authors:** Sergio Cavalieri, Marco Spinetta, Domenico Zagaria, Marta Franchi, Giulia Lavazza, Floriana Nardelli, Alessandro Serafini, Riccardo Leone, Antonio Messina, Francesco Arpaia, Giorgio Buccimazza, Serena Carriero, Felice D’Angelo, Elvira Stellato, Giulio Giuri, Maurizio Balbi, Giulia Chiara Preziosa, Margherita Parolise, Carlotta Pessina, Silvio Romano, Marco Curti, Davide Capra

**Affiliations:** 1 Postgraduate School in Radiodiagnostics, Università del Piemonte Orientale, Novara, Italy; 2 Postgraduate School in Radiodiagnostics, Università degli Studi di Torino, Torino, Italy; 3 Postgraduate School in Radiodiagnostics, Università Vita-Salute San Raffaele, Milano, Italy; 4 Postgraduate School in Radiodiagnostics, Università degli Studi di Milano, Milano, Italy; 5 Postgraduate School in Radiodiagnostics, Università degli Studi di Pavia, Pavia, Italy; 6 Postgraduate School in Radiodiagnostics, Università degli Studi Milano-Bicocca, Milano, Italy; 7 Postgraduate School in Radiodiagnostics, Università degli Studi di Brescia, Brescia, Italy; 8 Postgraduate School in Radiodiagnostics, Humanitas University, Pieve Emanuele, Italy; 9 Postgraduate School in Radiodiagnostics, Università degli Studi dell’Insubria, Varese, Italy; 10 Department of Biomedical Sciences for Health, Università degli Studi di Milano, Via Mangiagalli 31, 20133 Milano, Italy

**Keywords:** Survey and questionnaires, COVID-19, Pandemic, Education, distance, Radiology

## Abstract

**Objectives:**

To assess changes in working patterns and education experienced by radiology residents in Northwest Italy during the COVID-19 pandemic.

**Methods:**

An online questionnaire was sent to residents of 9 postgraduate schools in Lombardy and Piedmont, investigating demographics, changes in radiological workload, involvement in COVID-19-related activities, research, distance learning, COVID-19 contacts and infection, changes in training profile, and impact on psychological wellbeing. Descriptive and *χ*^2^ statistics were used.

**Results:**

Among 373 residents invited, 300 (80%) participated. Between March and April 2020, 44% (133/300) of respondents dedicated their full time to radiology; 41% (124/300) engaged in COVID-19-related activities, 73% (90/124) of whom working in COVID-19 wards; 40% (121/300) dedicated > 25% of time to distance learning; and 66% (199/300) were more involved in research activities than before the pandemic. Over half of residents (57%, 171/300) had contacts with COVID-19-positive subjects, 5% (14/300) were infected, and 8% (23/300) lost a loved one due to COVID-19. Only 1% (3/300) of residents stated that, given the implications of this pandemic scenario, they would not have chosen radiology as their specialty, whereas 7% (22/300) would change their subspecialty. The most common concerns were spreading the infection to their loved ones (30%, 91/300), and becoming sick (7%, 21/300). Positive changes were also noted, such as being more willing to cooperate with other colleagues (36%, 109/300).

**Conclusions:**

The COVID-19 pandemic changed radiology residents’ training programmes, with distance learning, engaging in COVID-19-related activities, and a greater involvement in research becoming part of their everyday practice.

**Key Points:**

• *Of 300 participants, 44% were fully dedicated to radiological activity and 41% devoted time to COVID-19-related activities, 73% of whom to COVID-19 wards.*

• *Distance learning was substantial for 40% of residents, and 66% were involved in research activities more than before the COVID-19 pandemic.*

• *Over half of residents were exposed to COVID-19 contacts and less than one in twenty was infected.*

**Supplementary Information:**

The online version contains supplementary material available at 10.1007/s00330-021-07740-0.

## Introduction

When the World Health Organization declared the COVID-19 outbreak as a pandemic on March 11, 2020, the Italian Healthcare system was already facing significant challenges responding to the virus spreading [1]. Since February 19, 2020, up to July 23, Italy has registered over 245,000 positive cases, most of which in northern regions [2]. Soon the Italian government put in place emergency public health measures to limit COVID19 spreading and to reduce the pressure on the healthcare system, by introducing security measures such as general lockdowns and social distancing [3].

Lombardy and Piedmont, being among the most COVID-19-affected Italian regions, have implemented restrictions on the provision of healthcare services to citizens, suspending deferrable and non-urgent hospitalisation and outpatient activities [4]. Likewise, radiology departments reshaped their work routine to better fit for the fight against the pandemic, while ensuring the safety of their workers: dedicated pathways for COVID-19-positive patients have been introduced, screening programs and non-urgent elective imaging have been postponed, while remote working has been encouraged whenever possible [5, 6].

Radiology training in Italy consists of a 4-year residency during which the residents rotate throughout the main radiological imaging modalities/techniques and subspecialties, gaining clinical experience in guiding the execution and in reporting diagnostic studies as well as in performing interventional procedures. In addition, they follow an educational programme mainly based on frontal lessons.

Due to the COVID-19 pandemic however, radiology residents had to abruptly change their personal and professional lives, facing high levels of stress [7, 8]. Part of them has been redeployed on a voluntary basis in intensive care units or in internal medicine wards [8]. Those who kept working in the radiology department faced changes in their work schedule, and the drop in both radiological workload and variety negatively affected their training. The COVID-19 pandemic pushed radiology teaching, once mainly based on in-person lectures, towards online education [8]. Postgraduate schools offered live or recorded online conferences; radiological societies, such as the European Society of Radiology and the Radiological Society of North America, made available their web-based educational platforms [9], offering webinars, courses, and clinical cases; the screen-sharing options during teleconferences made virtual side-by-side reporting possible. Finally, even on social media, platforms such as Radiopaedia (https://radiopaedia.org) shared educational content, in an immediate and user-friendly fashion. Residents were also encouraged to take part in research, focusing on COVID-19 pneumonia. The pandemic also impacted the psychological wellbeing of residents. First, they could be concerned to be infected and/or fear to expose their loved ones to the risk of illness. Indeed, a recent paper by Rainford et al showed that 87.8% of student radiographers fear the risk of infecting their families [10]. Such concern could be worsened by shortage or misuse of personal protective equipment (PPE) [8]. Second, abrupt changes in work schedules, prolonged working hours or, vice versa, prolonged periods of inactivity, could be powerful stressors. Third, social distancing, pivotal to contain the virus diffusion, could itself be psychologically challenging. Of note, a study by Cao et al [11] found that up to 25% of college students experienced symptoms of anxiety during the outbreak in the Hubei province in China.

Hence, given the profound impact of COVID-19 pandemic on radiology residents, we aimed to assess the changes in working patterns and education experienced by the residents of two of the most affected Italian regions, and the perceived impact on their psychological wellbeing.

## Materials and methods

### Study design and recipients

Ethics committee approval was not needed for this study, as all data was collected anonymously, and participation was voluntary. This survey was developed by S.C., M.S., and D.Z., residents at the postgraduate school in Radiodiagnostics of Università del Piemonte Orientale, who ideated the first draft of the questionnaire and established the areas of interest. Then, directors of each radiology postgraduate school in Lombardy and Piedmont reviewed the questionnaire to improve the focus on changes in residents’ learning activities and to ease data analysis, then approved the questionnaire. Finally, the questionnaire was pretested by a panel of seven residents and refined accordingly. The questionnaire was then published online on a dedicated software platform (Google Forms, Google). Subsequently, all radiology residents of Lombardy and Piedmont were invited to anonymously participate by the central office or the director of each school with an e-mail including a link to the survey form.

The self-administered questionnaire was available for 2 weeks, from May 7 to May 21, 2020. Two reminders were sent on May 13 and May 18. All participants had to give their consent to personal data treatment in order to access the questionnaire.

The survey consisted of 24 questions, two of which could be skipped depending on the answer to the previous question. An introductive group of questions focused on participants’ demographics (age, gender, residency year, location of postgraduate school). Subsequently, a further group assessed the perceived percentage changes in residents’ and departments’ daily workload caused by the COVID-19 pandemic, and changes in activities dedicated to distance learning. Two questions investigated the tools used for distance learning, and a personal forecast whether online learning tools will be more used after the outbreak resolution.

The following set of questions concerned the residents’ involvement in collaborations with other hospital departments or outside the hospital, expressed as a percentage of general activity. If the residents participated in any kind of activity outside the hospital, they were asked to specify the activity they were involved with. Subsequently, three questions relating to personal safety were formulated, assessing if the residents did come in close contact with confirmed COVID-19-positive subjects as defined by the European Centre for Disease Prevention and Control [12], and the percentage of observed good use of PPE in and outside their postgraduate school. Changes in both dedication and topics of research activity were investigated in the following questions, as well as changes in the importance attributed to thoracic imaging during their training course. Two questions investigated if the residents were evaluating changes in their future subspecialty profile and whether they would pursue again the radiologist career, given the implications of the COVID-19 pandemic. The last group of questions assessed the impact of the COVID-19 outbreak on residents themselves, investigating whether they contracted the infection, if they experienced the loss of a loved one, and the perceived impact on their psychological state. The original questionnaire and an English translation are provided as Supplementary Material (ESM 1).

### Statistical analysis

After survey closure on May 21, 2020, results were exported in a spreadsheet for statistical analysis. Categorical variables were assumed to have a skewed distribution. The Shapiro-Wilk test was used to assess distribution of continuous variables. Thus, median and interquartile range (IQR) or mean and standard deviation were used according to data distribution. Descriptive statistics were expressed as absolute frequencies and percentages for categorical variables. Differences between variables were assessed by *χ*^2^ statistics; Cramer’s V was used to compute the magnitude of associations. Cramer’s V values were interpreted according to Cohen [13]. *P*-values < 0.05 were considered as significant. Statistical analysis was performed using R v3.5.3 for Windows (The R Foundation for Statistical Computing).

## Results

A total of 373 residents from nine postgraduate schools in Lombardy and Piedmont were invited to participate in the survey. The questionnaire was completed by 300 participants, 135 (45.0%) of whom women, resulting in an overall 80.4% response rate. The percentage of residents from each postgraduate school and participants’ demographic are reported in Table 1. To account for imbalanced classes, only males and females were included in *χ*^2^ gender analysis. The median age of participants was 29 years (interquartile range 27–30 years), residents were divided into junior residents (first and second year of residency) and senior residents (third, fourth, and fifth year of residency).

**Table 1:** Participants’ characteristics

	*n*	%	Response rate* (%)
Gender			
Female	135	45.0	
Male	155	51.7	
Transgender	1	0.3	
Prefer not to answer	9	3.0	
Residency year			
First	95	31.7	
Second	72	24.0	
Third	74	24.7	
Fourth	58	19.3	
Fifth	1	0.3	
Affiliation			
Università del Piemonte Orientale	32	10.7	100.0
Università degli Studi di Torino	72	24.0	97.3
Università Vita-Salute San Raffaele	24	8.0	96.0
Università degli Studi di Milano	80	26.7	82.5
Università degli Studi di Pavia	30	10.0	76.9
Università degli Studi di Milano-Bicocca	25	8.3	64.1
Università degli Studi di Brescia	21	7.0	58.3
Humanitas University	9	3.0	56.3
Università degli Studi dell'Insubria	7	2.3	38.9

*Response rate from each postgraduate school

### Radiology department activity

**Fig. 1:**
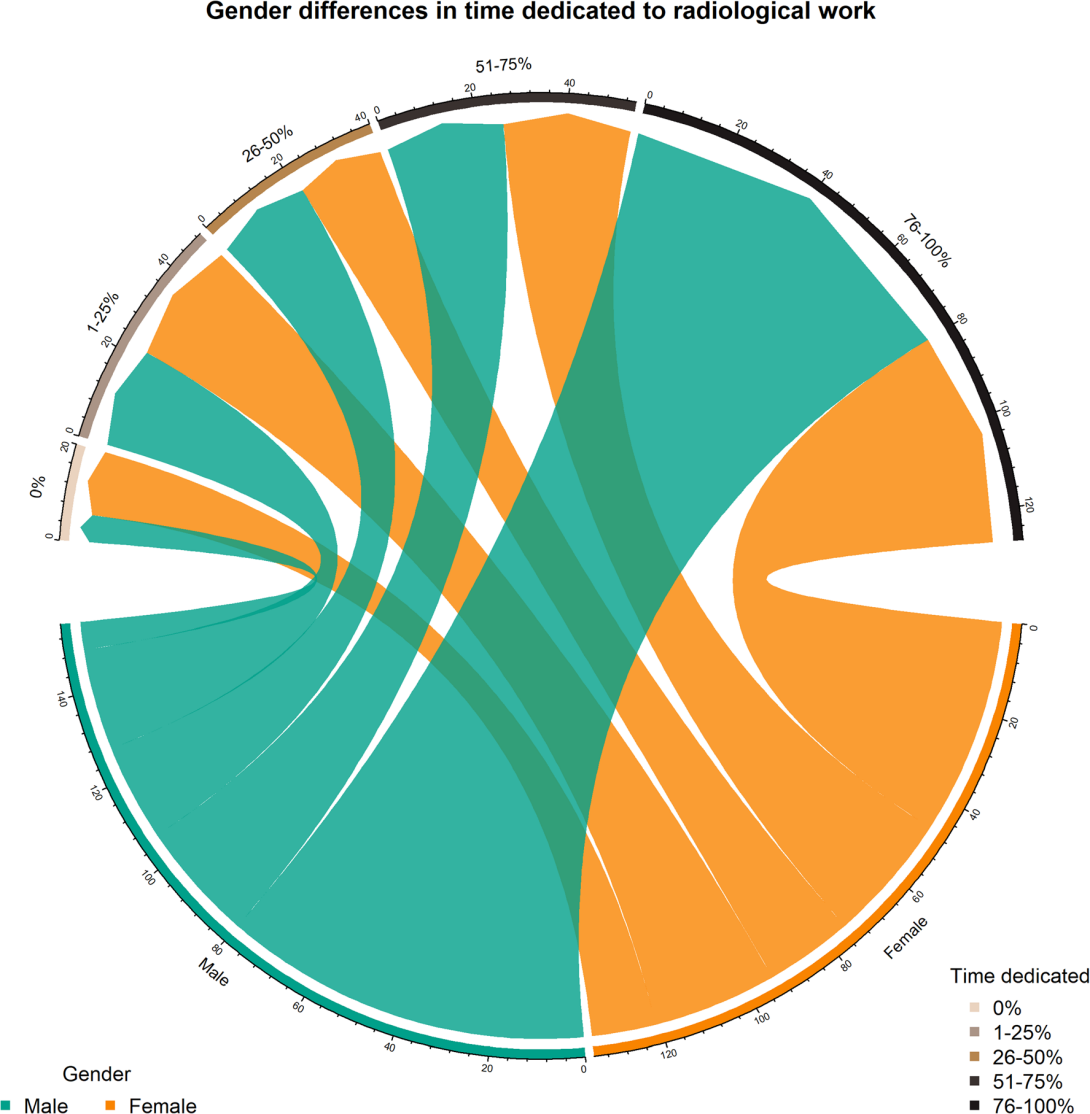
Chord diagram outlining gender differences in time dedication to radiological work during the pandemic emergency. Each arrow represents the number of residents dedicating the designated percentage of perceived working time to radiological work. Note the relative gender disproportions in residents not dedicated and fully dedicated to radiological work

When asked to evaluate the perceived workload reduction in the radiology department they were assigned, 43.3% (130/300) residents declared a reduction in the 40–59% range, while 33.3% (100/300) noted a reduction over 60% and 23.3% (70/300) under 40%. Senior residents were moderately more likely to report a perceived reduction in radiological workload over 60% than junior residents (39.8%, 53/133 *versus* 28.1%, 47/167, *p* = 0.016, Cramer’s V = 0.215). Nonetheless, 44.3% (133/300) of residents were fully dedicated to the radiology service, 18.3% (55/300) dedicated 51–75% of their time, and 37.3% (112/300) dedicated less than 50% of their time, without significant differences among years of residency (*p* = 0.170). Female residents were relatively more likely to dedicate less time to the radiology service than males (Fig. 1): 10.4% (14/135) reported not dedicating any time and 34.8% (47/135) dedicating their full time, compared with 3.9% (6/155) and 51.6% (80/155) of males not dedicated and fully dedicated, respectively (*p* = 0.025, Cramer’s V = 0.196).

### Distance learning and research activity

**Fig. 2:**
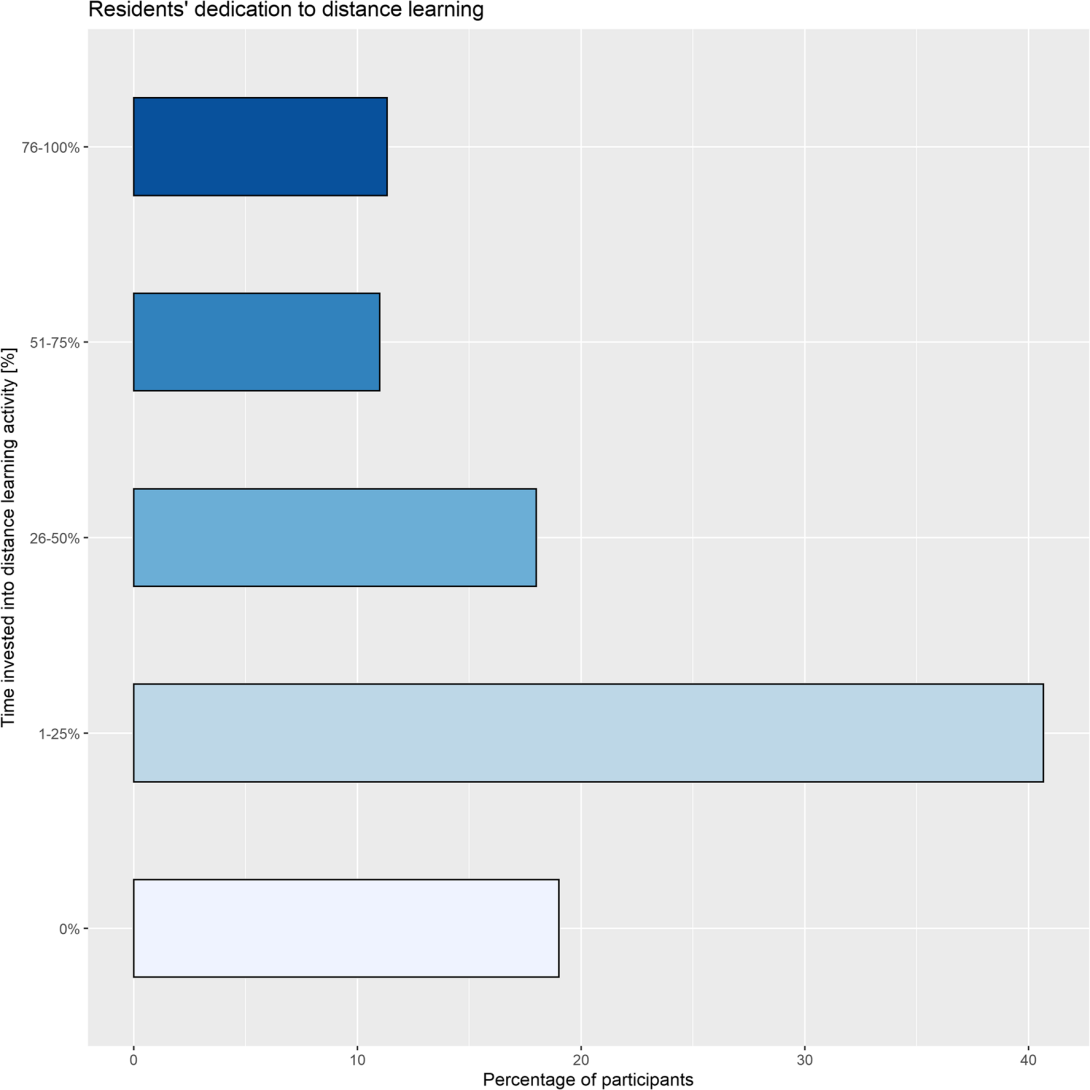
Residents’ dedication to distance learning

**Fig. 3:**
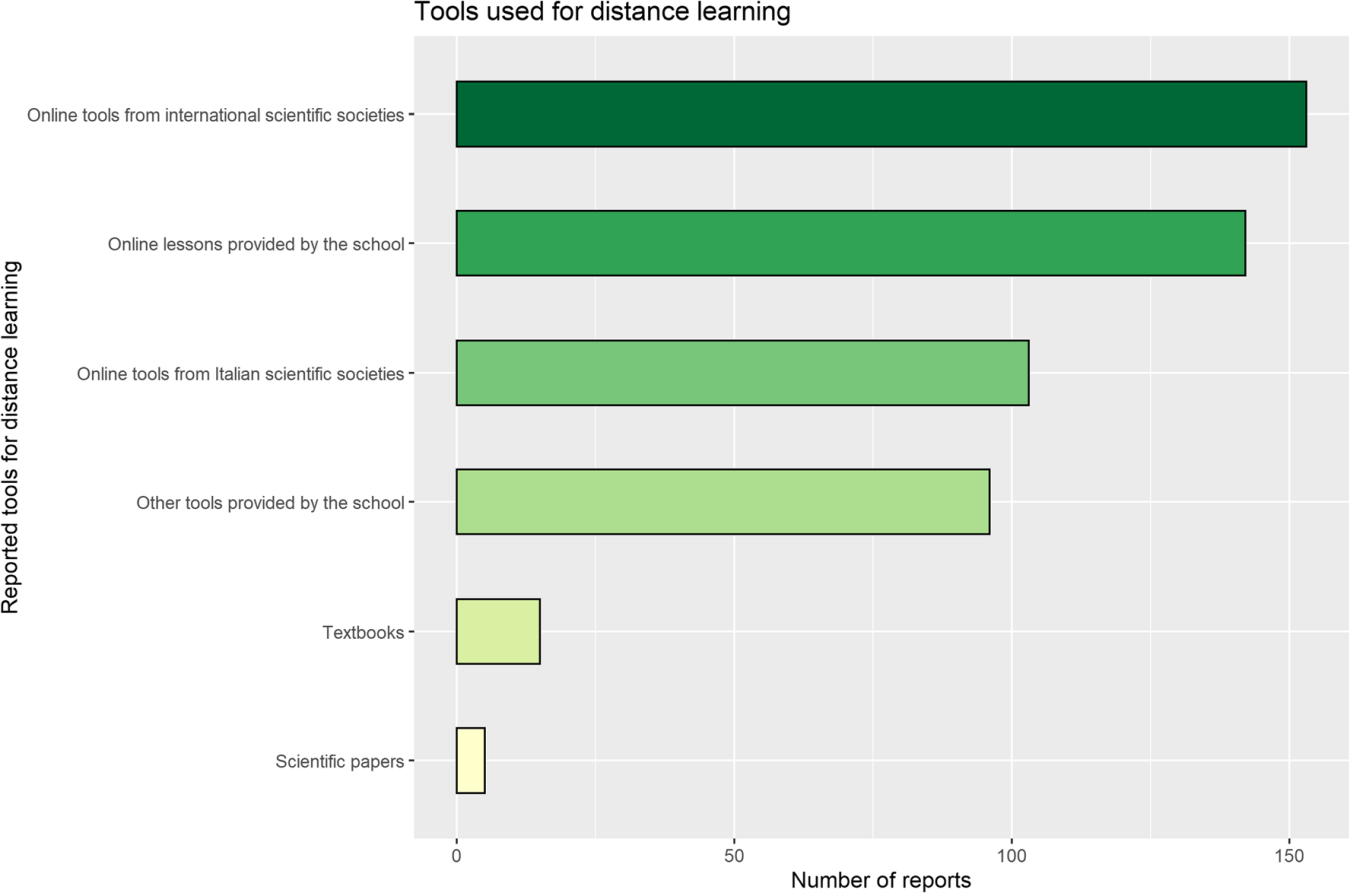
Tools used by residents for distance learning

During the pandemic peak, 40.3% (121/300) of residents invested over 25% of their time in distance learning activities. Figure 2 summarises participants’ dedication to distance learning. A bigger proportion of senior residents was fully dedicated to distance learning than junior residents (18.8%, 25/133 *versus* 5.4%, 9/167, *p* = 0.006, Cramer’s V = 0.221). No significant differences were found by gender (*p* = 0.465). The most common tools for distance learning were online tools from international scientific societies (51.0%, 153/300) (Fig. 3).

Among the residents, 73.0% (219/300) answered that online learning tools will be more used after the pandemic (Table 2). Senior residents were less likely to notice changes in the importance given to thoracic imaging: 28.6% (38/133) of them did not report any variation compared with 15.0% (25/167) of junior residents (*p* = 0.009, Cramer’s V = 0.175). No significant differences were found by gender (*p* = 0.168).

**Table 2:** Future perspectives on online learning, importance given to thoracic imaging, and involvement in research activities

	*n*	%
Do you feel that online learning tools will be used more after the COVID-19 pandemic?		
Definitely yes	40	13.3
Probably yes	179	59.7
I don’t know	39	13.0
Probably not	36	12.0
Definitely not	6	2.0
Did the COVID-19 change the importance given to thoracic imaging in your education?		
Mild increase in the importance given to thoracic imaging	161	53.7
Marked increase in the importance given to thoracic imaging	76	25.3
No changes	63	21.0
During the pandemic emergency, your research activity has seen:		
A mild increase	92	30.7
A marked increase	74	24.7
A steep increase	33	11.0
No changes	101	33.7
The topics of your research activity:		
Had some changes in relation to COVID-19	93	31.0
Had marked changes in relation to COVID-19	63	21.0
All my research focused on COVID-19	39	13.0
Did not change	105	35.0

The pandemic emergency increased the participation of 66.3% (199/300) residents in research activities. Similarly, research topics changed, with 65.0% (195/300) of residents reporting a parallel shift towards COVID-19-related research. Senior residents were less likely to focus on COVID-19-related research than junior residents: 42.1% (56/133) of senior residents *versus* 29.3% (49/167) of junior residents did not report any significant changes in research topics (*p* = 0.003, Cramer’s V = 0.215). No significant differences were found by gender (*p* = 0.903).

### COVID-19-related activities

Overall, 41.3% (124/300) of residents choose to devote part of their time to COVID-19-related activities, 72.6% (90/124) of whom was engaged in COVID-19 wards. No differences were found by gender or residency seniority (*p* > 0.130). Details about residents’ commitment against COVID-19 are reported in Table 3.

**Table 3:** Residents’ COVID-19-related activity

	*n*	%
How much of your activity was dedicated to collaborations with COVID-19 wards?		
0%	210	70.0
1–25%	53	17.7
26–50%	17	5.7
51–75%	12	4.0
76–100%	8	2.7
How much of your activity was dedicated to COVID-19-related activities outside the hospital?		
0%	247	82.3
1–25%	27	9.0
26–50%	15	5.0
51–75%	9	3.0
76–100%	2	0.7
COVID-19-related activities in and outside the hospital:		
Hospital extra-radiological support activities (*e.g.* triage at hospital entrance)	25	8.3
Services of primary care	12	4.0
Volunteering	4	1.3
Telemedicine services	3	1.0

### Preventive measures and COVID contacts and infections

When asked to rate the percentage of correct usage of PPE, hygienic preventive measures, and social distancing in their work environment, most residents (66.7%, 200/300) observed correct preventive behaviours in over 60% of the cases in the postgraduate schools’ structures. However, outside the postgraduate schools’ structure, the majority of residents reported a correct application of preventive measures in less than 59% of the cases (54.0%, 162/300) (Table 4).

**Table 4:** Percentage of observed correct usage of safety measures for preventing infection

	*n*	%
How would you rate the percentage of correct use of PPE, social distancing, and hygienic safety measures in your school's structures?		
0%	2	0.7
1–10%	7	2.3
11–39%	21	7.0
40–59%	70	23.3
60–89%	121	40.3
90–99%	70	23.3
100%	9	3.0
How would you rate the percentage of correct use of PPE, social distancing, and hygienic safety measures outside your school's structures, both in and out-of-hospital settings?		
0%	13	4.3
1–10%	11	3.7
11–39%	43	14.3
40–59%	95	31.7
60–89%	111	37.0
90–99%	23	7.7
100%	4	1.3

**Fig. 4:**
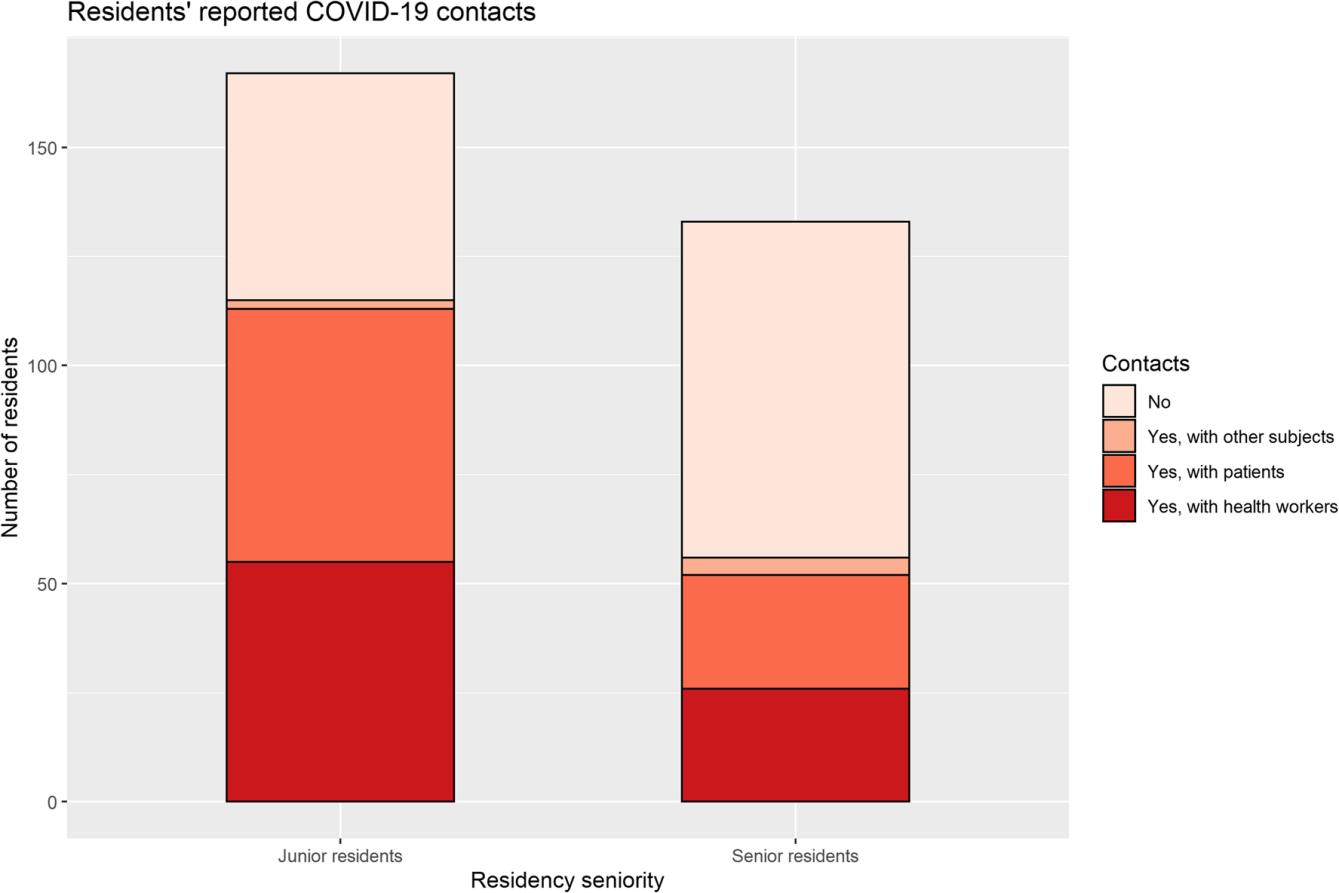
Proportions of junior and senior residents reporting COVID-19 contacts

Overall, most residents (57.0%, 171/300) had contacts with COVID-19-positive subjects. Senior residents were disproportionately less exposed to COVID-19 contacts than junior residents. In fact, 57.9% of them (77/133) did not have any COVID-19 contact, compared with 31.1% (52/167) of junior residents not reporting contacts (*p* < 0.001, Cramer’s V = 0.286) (Fig. 4). No significant differences were found by gender (*p* = 0.081). Details about residents’ COVID-19 contacts and infection are provided in Table 5.

**Table 5:** Residents’ COVID-19 contacts and infection

	*n*	%
Did you have close contacts with COVID-19-positive subjects?		
No	129	43.0
Yes, with patients	84	28.0
Yes, with health workers	81	27.0
Yes, with other subjects	6	2.0
Did you contract the SARS-CoV-2 infection?		
No, I never had symptoms and never have been tested	106	35.3
No, I tested negative in a serological test	93	31.0
No, I tested negative in one or more RT-PCR tests	75	25.0
Unsure	9	3.0
Maybe, I was symptomatic but had one or more negative RT-PCR tests	3	1.0
Yes, tested positive at a serological test and negative at the RT-PCR test	5	1.6
Yes, symptomatic but never tested	3	1.0
Yes, paucisymptomatic with a positive RT-PCR test	2	0.7
Yes, paucisymptomatic with a positive serological test	2	0.7
Yes, symptomatic with a positive RT-PCR test	1	0.3
Yes, symptomatic with a positive serological test	1	0.3

*RT-PCR*, reverse transcriptase polymerase chain reaction

Less than 5% (4.7%, 14/300) of residents were infected, 91.3% (274/300) were not, and 4.0% (12/300) were uncertain regarding their status. Compared with junior residents, senior residents were remarkably less tested for infection: 48.1% (64/133) of senior residents *versus* 25.1% (42/167) of junior residents never had symptoms nor had undergone serological or reverse transcriptase polymerase chain reaction (RT-PCR) tests. Moreover, 32.9% (55/167) of junior residents *versus* 12.8% (17/133) of senior residents tested negative on one or more RT-PCR tests (*p* < 0.001, Cramer’s V = 0.403). No significant differences were found by gender (*p* = 0.468). During the pandemic emergency, 7.7% (23/300) of residents experienced the loss of a loved one.

### Psychological impact and future directions

Only 1.0% (3/300) of residents stated that, given the implications of this pandemic scenario, they would not have chosen radiology as their specialty and 7.3% (22/300) would change their subspecialty. Senior residents were notably more determined than junior residents in pursuing the subspecialty of their choice, 27.1% (36/133) would firmly refuse to change subspecialty compared with 10.2% (17/167) of junior residents, and 46.7% (78/167) of junior residents had yet to choose a subspecialty, compared with 18.8% (25/133) of senior residents in the same situation (*p* < 0.001, Cramer’s V = 0.334).

The most common concerns affecting residents’ psychological wellbeing were spreading the infection to their loved ones (30.3%, 91/300) and becoming sick (7.0%, 21/300). Positive changes were also noted, such as being more willing to cooperate with other colleagues (36.3%, 109/300). Nonetheless, 47.7% (143/300) of residents reported that the COVID-19 pandemic did not affect their psychological status.

## Discussion

Just over 80% (80.4%, 300/373) of Lombardy and Piedmont radiology residents answered our survey, a result that suggests the residents were eager to share their experience during this life-changing emergency.

This survey revealed that over 40% of radiology residents devoted part of their time to COVID-19-related activities, pointing out how a consistent amount of radiology residents consider themselves first and foremost “physicians,” and highlights the cross-disciplinary potential of radiology training. As noted by one of the respondents in the free text comments, radiologists cannot prescind from correlating the images to the clinical status of the patient, favouring a holistic approach to the patient. Furthermore, despite a marked reduction in the radiology departments’ daily workload according to most residents, the majority of them kept working in the department, although on reduced shifts in 49% of the cases. Residents, though, were aware of the risks connected with their choice of redeploying in COVID-19-related activities or to keep working in radiology departments: over one-third of them were concerned about becoming infected and/or communicating the disease to their loved ones. During their activity, 57% of residents were exposed to COVID-19 contacts and less than 5% become infected.

A small but significant difference in dedication to radiologic work during the pandemic emergency between male and female residents was found. This might be a reflection of the gender gap that still persists on the balance between work and family care: the burden of familiar care is weighting mostly on women’s shoulders, pushing them to reduce the working hours [14]. This could have been particularly true under the general lockdown enforced by Italian authorities, where schools and daycare institutions were closed, forcing families to take charge of care work that was until then externalised.

Unsurprisingly, distance learning has been substantial for over 40% of residents. This is in keeping with the existing reports on the impact of the pandemic on radiology residency programmes [7, 8, 15, 16] that identified off-site learning as crucial for the continuity of radiological education. Nonetheless, almost 20% of the participants to this survey never dedicated time to distance learning, probably because of engagement in other activities. Regarding the tools used for distance learning, an overwhelming preference went to online courses, webinars, and lessons offered by scientific societies and postgraduate schools, although textbooks were still used by 5% of residents. Notably, only one resident was concerned about the impact that the reduction in deferrable radiologic procedures could have on her/his education, in contrast with previous anecdotal reports [7, 16]. Moreover, about two-thirds of residents were more involved in research activities than before the COVID-19 pandemic, showing how a clinical emergency sparked the interest in scientific research, especially in the presence of reduced radiological workload.

A persistent difference between junior and senior (first 2 and last 3 years of residency respectively) residents was found: senior residents were more likely to report a more marked reduction in radiological workload, to do more distance learning, kept focusing on their previous research topics, were less exposed to COVID-19 contacts, and consequently were less tested for infection. The root cause of this discrepancy might be related to the structure of training pathways during residency: senior residents in fact, after training in general radiology during junior years, may have chosen some subspecialties, sometimes taking part in hyper-specialist research projects. Those subspecialties, though, have seen their exam numbers drop far more than general radiology, consisting mostly of those deferrable procedures that have been in most cases postponed [4–6].

The main limitation of this survey is the lack of a detailed assessment of the radiological learning curve during the COVID-19 emergency. We could argue that good standards were anyway maintained: only one resident was concerned about her/his professional education and 99% of residents confirmed their preference for radiology. Nevertheless, the absence of specific questions hinders any possible conclusion about teaching quality during the COVID-19 emergency. A follow-up survey could allow the evaluation of long-term outcomes of radiology residents’ training after the COVID-19 pandemic. Besides, international studies could provide insights into how differences among the management of COVID-19 pandemic in several countries and different residency structures may have affected radiology residents. Moreover, we assessed the residents’ self-perceived psychological wellbeing through a single question. Further studies could deeper assess the psychological impact of the COVID-19 pandemic on radiology residents using validated resilience scores.

Finally, our questionnaire did not include cognitive pre-test components, nor has been tested for re-test reliability, possibly limiting the reproducibility of the results of our questionnaire. In addition to that, this study is partially limited by the different response rate among the postgraduate schools, ranging from 100 to 39%. Schools with fewer residents might be affected by low response rates and be underrepresented.

In conclusion, this survey illustrated that in Lombardy and Piedmont the COVID-19 abruptly changed radiology residents’ activities. Distance learning became essential for 40% of residents, replacing in-person lessons, and about two-thirds of residents became more involved than before in research activities. Over 40% of residents engaged in COVID-19-related activities, and less than one in twenty was infected. Despite the subversion of their training programme, 99% of residents confirmed the preference for radiology.

## Supplementary information

ESM 1 (DOCX 36 kb)

## Abbreviations

PPE: Personal protective equipment

## Declarations

### Guarantor

The scientific guarantor of this publication is Davide Capra, MD.

### Statistics and biometry

No complex statistical methods were necessary for this paper.

### Methodology

• cross-sectional study
