# Supplementary material for: The impact of COVID-19 pandemic on radiology residents in Northern Italy
Source: Eur Radiol. 2021 Mar 23;31(9):7077–87. doi: 10.1007/s00330-021-07740-0 (PMC7986178; doi:10.1007/s00330-021-07740-0)
Supplement: Supplementary file 1 — (DOCX 36 kb) [file 330_2021_7740_MOESM1_ESM.docx]

**ESM 1 Original questionnaire and its translation, parallel text, Italian and English**

| **Ventiquattro domande agli Specializzandi**  **in Radiodiagnostica delle Scuole**  **lombarde e piemontesi sull’esperienza**  **COVID-19** | **Twenty-four questions to radiodiagnostics residents of Lombardy and Piedmont postgraduate schools on the COVID-19 experience** |
| --- | --- |
| * Campo obbligatorio | * Mandatory answer |
| 1. Consenso al trattamento dei dati *   Come previsto dalle attuali normative vigenti (legge 31 dicembre 1996 n.675, 676 Gazzetta Ufficiale del 08/01/1997, art.7 del D. Lgs. 30 giugno 2003, n.196 e Regolamento Europeo sulla Privacy UE 2016/679, General Data Protection Regulation - GDPR) sul trattamento dei dati personali e sul rispetto della privacy, i suoi dati verranno trattati in modo rigorosamente anonimo. Non le saranno richiesti dati inerenti alla Sua identità e le risposte fornite non saranno riconducibili a lei. Le informazioni che ci fornirà verranno trattate con la massima riservatezza, nel pieno rispetto delle leggi sulla privacy, in forma aggregata insieme a quelle di tutti gli altri partecipanti ed utilizzate solo a fini di studio e di ricerca.   - Do il consenso - Nego il consenso | 1. Consent to data processing *   In compliance with current legislation (law of 31 December 1996 n.675, 676 Gazzetta Ufficiale of 08/01/1997, art.7 of legislative decree 30 June 2003, n.196, and European Regulation UE 2016/679, General Data Protection Regulation – GDPR) on personal data processing and privacy, your data will be elaborated strictly anonymously. We will not ask data inherent your identity and the given answers will not be attributable to you. The data you will provide will be treated with the utmost confidentiality, in full respect of current privacy legislation, and in aggregated form together with all other participants’ data and used only for research purposes.   - I consent - I do not consent |
| 1. Quanti anni hai? *   _______ anni | 1. How old are you? *   _______ years |
| 1. Genere *  - Preferisco non rispondere - Donna - Uomo - Altro: _______ | 1. Gender *  - Prefer not to answer - Female - Male - - Other: _______ |
| 1. Anno di corso *  - Primo - Secondo - Terzo - -Quarto - Quinto | 1. Residency year *  - First - Second - Third - Fourth - Fifth |
| 1. Ateneo *  - Brescia - Milano Bicocca - Milano Humanitas - Milano Statale - Milano Vita-Salute - Pavia - Piemonte Orientale - Torino - Varese | 1. University *  - Brescia - Milano Bicocca - Milano Humanitas - Milano Statale - Milano Vita-Salute - Pavia - Piemonte Orientale - Torino - Varese |
| 1. Durante l'emergenza pandemica (marzo e aprile 2020), quanto della tua attività complessiva è stata dedicata alla frequenza al Servizio di Radiologia al quale eri assegnato? *  - 0% - 1–25% - 26–50% - 51–75% - 76–100% | 1. During the pandemic emergency (March and April 2020), how much of your activity was dedicated to radiology service? *  - 0% - 1–25% - 26–50% - 51–75% - 76–100% |
| 1. Durante l'emergenza pandemica (marzo e aprile 2020), valuti che la riduzione globale dell’attività del Servizio di Radiologia al quale eri assegnato sia stata del…? *  - 0% - 1–10% - 11–39% - 40–59% - 60–89% - 90–99% - 100% | 1. During the pandemic emergency (March and April 2020), how would you rate the reduction of the total daily workload in your department? *  - 0% - 1–10% - 11–39% - 40–59% - 60–89% - 90–99% - 100% |
| 1. Hai invece rilevato un incremento globale valutabile intorno al ___%? *   _______ | 1. Did you instead perceived an increase of around ____%? *   _______ |
| 1. Durante l'emergenza pandemica (marzo e aprile 2020), quanto della tua attività complessiva è stata dedicata alla formazione a distanza (FAD) da casa? *  - 0% - 1–25% - 26–50% - 51–75% - 76–100% | 1. During the pandemic emergency (March and April 2020), how much of your activity was dedicated to distance learning? *  - 0% - 1–25% - 26–50% - 51–75% - 76–100% |
| 1. Se hai condotto attività FAD, quali strumenti hai utilizzato? *   *Seleziona tutte le voci applicabili*   - Lezioni on-line o registrate fornite Scuola - Altro materiale fornito dalla Scuola - Link a siti di società scientifiche italiane - Link a società scientifiche internazionali - Altro: _______ | 1. Which tools did you use for distance learning? *   *Select all pertinent answers*   - Online or recorded lessons provided by the School - Other material provided by the School - Links to Italian scientific societies websites - Links to international scientific societies websites - Other: _______ |
| 1. Ritieni che in futuro, dopo la pandemia, la didattica online sarà molto più utilizzata rispetto al periodo precedente la pandemia? *  - Decisamente si - Probabilmente si - Non so - Probabilmente no - Decisamente no | 1. Will online learning tools be used more after the COVID-19 pandemic? *  - Definitely yes - Probably yes - I don’t know - Probably not - Definitely not |
| 1. Durante l'emergenza pandemica (marzo e aprile 2020), quanto della tua attività complessiva è stata dedicata alla collaborazione in reparti ospedalieri COVID-19? *  - 0% - 1–25% - 26–50% - 51–75% - 76–100% | 1. During the pandemic emergency (March and April 2020), how much of your activity was dedicated to collaborations with COVID-19 wards? *  - 0% - 1–25% - 26–50% - 51–75% - 76–100% |
| 1. Durante l'emergenza pandemica (marzo e aprile 2020), quanto della tua attività complessiva è stata dedicata ad attività extra-ospedaliere COVID-19-correlate: *  - 0% - 1–25% - 26–50% - 51–75% - 76–100% | 1. During the pandemic emergency (March and April 2020), how much of your activity was dedicated to COVID-19-related activities outside the hospital? *  - 0% - 1–25% - 26–50% - 51–75% - 76–100% |
| 1. Se non hai risposto 0% alla domanda precedente, a quali delle seguenti attività hai partecipato? *   *Seleziona tutte le voci applicabili*   - Attività di supporto extra-radiologiche presso l’ospedale nel quale si svolgeva la tua turnazione (esempio: triage all’entrata dell’ospedale) - Contratto presso ospedale al di fuori della rete formativa della Scuola - Volontariato presso strutture pubbliche - Volontariato presso strutture no-profit - Altro: _______ | 1. If you did not answer 0% at the previous question, in which of the following activities did you took part? *   *Select all pertinent answers*   - In-hospital extra-radiological support activities (*e.g.* triage at hospital entrance) - Contract with an hospital outside of the School’s structures - Volunteering in public organizations - Volunteering in nonprofit organizations - Other: _______ |
| 1. Hai avuto contatti stretti^†^ con soggetti COVID-19 positivi? *   ^†^Definizione di contatto stretto: 1) una persona che vive nella stessa casa di un caso di COVID-19; 2) una persona che ha avuto un contatto fisico diretto con un caso di COVID-19 (per esempio la stretta di mano); 3) una persona che ha avuto un contatto diretto non protetto con le secrezioni di un caso di COVID-19 (ad esempio toccare a mani nude fazzoletti di carta usati); 4)una persona che ha avuto un contatto diretto (faccia a faccia) con un caso di COVID-19, a distanza minore di 2 metri e di durata maggiore a 15 minuti; 5) una persona che si è trovata in un ambiente chiuso (ad esempio aula, sala riunioni, sala d'attesa dell'ospedale) con un caso di COVID-19 per almeno 15 minuti, a distanza minore di 2 metri; 6) un operatore sanitario od altra persona che fornisce assistenza diretta ad un caso di COVID19 oppure personale di laboratorio addetto alla manipolazione di campioni di un caso di COVID-19 senza l’impiego dei DPI raccomandati o mediante l’utilizzo di DPI non idonei; 7) una persona che abbia viaggiato seduta in aereo nei due posti adiacenti, in qualsiasi direzione, di un caso di COVID-19, i compagni di viaggio o le persone addette all’assistenza e i membri dell’equipaggio addetti alla sezione dell’aereo dove il caso indice era seduto (qualora il caso indice abbia una sintomatologia grave od abbia effettuato spostamenti all’interno dell’aereo, determinando una maggiore esposizione dei passeggeri, considerare come contatti stretti tutti i passeggeri seduti nella stessa sezione dell’aereo o in tutto l’aereo). Fonte: European Center for Disease Prevention and Control (ECDC), Tracciamento dei contatti: gestione sanitaria pubblica dei soggetti, inclusi gli operatori sanitari, entrati in contatto con casi di Covid-19 nell’Unione europea, 25 February 2020   - No - Si, con pazienti - Si, con personale sanitario - Si, con altri soggetti | 1. Did you have close contacts^†^ with COVID-19 positive subjects? *   ^†^Close contact definition: 1) A person living in the same household as a COVID-19 case 2) A person having had direct physical contact with a COVID-19 case (e.g. shaking hands) 3) A person having unprotected direct contact with infectious secretions of a COVID-19 case (e.g. being coughed on, touching used paper tissues with a bare hand) 4) A person having had face-to-face contact with a COVID-19 case within 2 metres and > 15 minutes 5) A person who was in a closed environment (e.g. classroom, meeting room, hospital waiting room, etc.) with a COVID-19 case for 15 minutes or more and at a distance of less than 2 metres 6) A healthcare worker or other person providing direct care for a COVID-19 case, or laboratory workers handling specimens from a COVID-19 case without recommended personal protective equipment (PPE) or with a possible breach of PPE 7) A contact in an aircraft sitting within two seats (in any direction) of the COVID-19 case, travel companions or persons providing care, and crew members serving in the section of the aircraft where the index case was seated (if severity of symptoms or movement of the case indicate more extensive exposure, passengers seated in the entire section or all passengers on the aircraft may be considered close contacts). Source: European Center for Disease Prevention and Control (ECDC), Contact tracing: Public health management of persons, including healthcare workers, having had contact with COVID-19 cases in the European Union, 25 February 2020   - No - Yes, with patients - Yes, with health workers - Yes, with other subjects |
| 1. Nella tua esperienza presso le strutture della Scuola di specializzazione, quale percentuale di corretto utilizzo dei dispositivi di protezione individuale (DPI) e di rispetto delle norme igieniche e di distanziamento sociale valuti sia avvenuta? *  - 0% - 1–10% - 11–39% - 40–59% - 60–89% - 90–99% - 100% | 1. In your experience, how would you rate the percentage of correct use of personal protective equipment (PPE), social distancing and hygienic safety measures in the school's structures? *  - 0% - 1–10% - 11–39% - 40–59% - 60–89% - 90–99% - 100% |
| 1. Nella tua eventuale esperienza all’esterno della Scuola di Specializzazione, sia in ambito ospedaliero che extra-ospedaliero, quale percentuale di corretto utilizzo dei DPI e di rispetto delle norme igieniche e di distanziamento sociale valuti sia avvenuta *  - 0% - 1–10% - 11–39% - 40–59% - 60–89% - 90–99% - 100% | 1. In your experience, how would you rate the percentage of correct use of PPE, social distancing and hygienic safety measures outside the school's structures, both in and out-of-hospital settings? *  - 0% - 1–10% - 11–39% - 40–59% - 60–89% - 90–99% - 100% |
| 1. Durante l'emergenza pandemica (marzo e aprile 2020), la tua attività di ricerca è… *  - Rimasta invariata - Ha subito un incremento lieve - Ha subito un incremento marcato - Ha subito un incremento forte | 1. During the pandemic emergency (March and April 2020), your research activity has seen: *  - No changes - A mild increase - A marked increase - A steep increase |
| 1. Durante l'emergenza pandemica (marzo e aprile 2020), i temi della tua attività di ricerca… *  - Non hanno subito modificazioni - Hanno subito alcune modificazioni in relazione alla pandemia - Hanno subito notevoli modificazioni in relazione alla pandemia - Tutta l’attività di ricerca si è focalizzata sulla pandemia | 1. During the pandemic emergency (March and April 2020), the themes of your research activity: *  - Did not change - Had some changes in relation to COVID-19 - Had marked changes in relation to COVID-19 - All my research focused on COVID-19 |
| 1. Ritieni che la pandemia abbia comportato variazioni nel tuo percorso formativo relativamente all’importanza attribuita all’imaging toracico? *  - Nessuna variazione - Modesto incremento dell’importanza attribuita all’imaging toracico - Notevole incremento dell’importanza attribuita all’imaging toracico | \| 1. Has the COVID-19 changed the importance given to thoracic imaging in your education? *  - No changes - Mild increase in the importance given to thoracic imaging - Marked increase in the importance given to thoracic imaging \| \| --- \| |
| 1. Hai contratto l’infezione da SARS-CoV-2? *  - No, ho eseguito uno o più tamponi negativi - No, ho seguito esami sierologici negativi - No, mai avuto sintomi, non eseguiti test - Si, asintomatico con tampone positivo - Si, paucisintomatico con tampone positivo - Si, sintomatico con tampone positivo - Penso di sì: sintomatico con uno o più tamponi negativi - Altro: _______ | 1. Have you contracted the SARS-CoV-2 infection? *  - No, I tested negative in one or more RT-PCR tests - No, I tested negative in a serological test - No, I never had symptoms and never have been tested - Yes, asymptomatic with a positive RT-PCR test - Yes, paucisymptomatic with a positive RT-PCR test - Yes, symptomatic with a positive RT-PCR test - Maybe, I was symptomatic but had one or more negative RT-PCR tests - Other: _______ |
| 1. La pandemia COVID-19 ha determinato, almeno temporaneamente, mutamenti nel peso relativo e nella rilevanza clinica dell’attività dei diversi ambiti iperspecialistici della nostra Scuola. Questo nuovo quadro ti porterà a modificare il profilo iperspecialistico che avevi programmato? *  - Non avevo ancora programmato uno specifico profilo iperspecialistico - Decisamente si - Probabilmente si - Non so - Probabilmente no - Decisamente no | 1. The COVID-19 pandemic has determined, at least temporarily, changes in the relative importance and clinical relevance of the activity of various subspecialties of the Radiodiagnostics School. This new context will drive you to change the subspecialty profile that you were pursuing? *  - I still have to choose a subspecialty profile - Definitely yes - Probably yes - I don’t know - Probably not - Definitely not |
| 1. La pandemia COVID-19 ha determinato mutamenti rilevanti dell’attività delle diverse specializzazioni mediche. In questo nuovo quadro, sceglieresti ancora la Scuola di Specializzazione in Radiodiagnostica? *  - Decisamente si - Probabilmente si - Non so - Probabilmente no - Decisamente no | 1. The COVID-19 pandemic has determined relevant changes in the activities of many different medical specialisations. In this new context, would you pursue again a specialisation in Radiodiagnostics? *  - Definitely yes - Probably yes - I don’t know - Probably not - Definitely not |
| 1. Come valuti l’impatto psicologico della pandemia su te stesso: *   *Seleziona tutte le voci applicabili*   - Mi sento più disponibile di prima a collaborare con i colleghi della radiologia - Mi sento più disponibile di prima a collaborare con i colleghi degli altri reparti - Il mio stato psicologico non è sostanzialmente cambiato - Il mio stato psicologico risente della preoccupazione per la possibilità di contrarre la malattia - Il mio stato psicologico risente della preoccupazione di infettarmi e trasmettere la malattia a persone a me care - Altro: _______ | 1. How do you evaluate the psychological impact of the pandemic on yourself?   *Select all pertinent answers*   - I feel more willing than before to cooperate with other colleagues from the radiology department - I feel more willing than before to cooperate with other colleagues from other departments - My psychological status was not affected - My psychological status is affected by the concern of becoming sick - My psychological status is affected by the concern of contracting the disease and infect my dear ones - Other: _______ |
| 1. Durante la pandemia hai avuto esperienza di persone a te care che sono purtroppo decedute a causa del COVID-19? *  - Si - No | 1. During the pandemic, did you suffer the loss of loved ones that sadly died of COVID-19? *  - Yes - No |
